# An Unusual Case of Hyperhemolysis Syndrome and Delayed Hemolytic Transfusion Reaction due to Anti-Jk(a) and Anti-P1 Antibodies

**DOI:** 10.1155/2023/5290115

**Published:** 2023-12-31

**Authors:** Hunter Montgomery, Matthew X. Luo, Steven Baker, Ming Y. Lim

**Affiliations:** ^1^University of Utah School of Medicine, Salt Lake City, UT, USA; ^2^Department of Pathology, University of Utah, Salt Lake City, UT, USA; ^3^Department of Transfusion Medicine, University of Utah, Salt Lake City, UT, USA; ^4^Division of Hematology & Hematologic Malignancies, Department of Internal Medicine, University of Utah, Salt Lake City, UT, USA

## Abstract

**Background:**

Hyperhemolysis syndrome (HS) is a severe hemolytic transfusion reaction that can cause hemoglobin and hematocrit levels to drop below pretransfusion levels, leading to severe anemia. HS most commonly occurs in patients with a pre-existing hemoglobinopathy such as sickle cell disease (SCD) or beta-thalassemia.

**Methods:**

We report a case of HS, occurring in the absence of hemoglobinopathy, making the diagnosis challenging. The patient reported was also affected by a CIC-rearranged sarcoma. As part of the workup, the patient received a bone marrow biopsy for suspected hemophagocytic lymphohistiocytosis.

**Results:**

This provided a rare biopsy specimen to correlate reticulocytopenia with marked erythroid hyperplasia in the marrow, supporting the hypothesis of reticulocyte destruction as a contributing cause of anemia in these patients. This patient had demonstrable alloantibodies to the Jk(a) and P1 antigens as potential triggers for HS.

**Conclusions:**

It is vital that a diagnosis of HS be correctly made in these patients with severe anemia, as blood transfusions generally lead to worsening of their conditions.

## 1. Case Presentation

A 42-year-old woman with newly diagnosed capicua transcriptional repressor (CIC)-rearranged sarcoma (CIC-DUX4 translocation positive) had recently completed cycle 1 of VAI (vincristine 2 mg, doxorubicin 750 mg/m^2^ with dexrazoxane, and ifosfamide 2000 mg/m^2^) at our institution and was discharged to home. Five days later, she presented to an outside institution with a hemoglobin (Hgb) of 6.4 g/dL. She received her first ever blood transfusion, which was 2 units of packed red blood cells (pRBCs). These were well tolerated and resulted in a posttransfusion Hgb of 8.0 g/dL. Nine days later, she presented to the same institution with chills, flu-like symptoms, headache, and dark amber-colored urine.

Dipstick urine was positive for blood (“large”) and 2+ for protein and negative for bilirubin, nitrites, ketones, and leukocyte esterase. Urine microscopic analysis revealed nine white blood cells and one red blood cell per high power field and trace bacteria. A computed tomography scan of her chest, abdomen, and pelvis was unremarkable. Laboratory findings showed an Hgb of 8.5 g/dL. She was diagnosed with a presumed urinary tract infection, received one dose of intravenous ceftriaxone, and was discharged home with seven days of cefalexin.

The following day, she presented again to the outside institution with excessive fatigue and reported passing blood in her urine. The complete blood count (CBC) showed an Hgb of 7.0 g/dL, a WBC of 2.96 K/uL, a platelet count of 17 × 10^9^/L, a mean corpuscular volume of 92 fL, and a red cell distribution width of 18.2%. A comprehensive metabolic panel (CMP) revealed mild hyponatremia at 132 mmol/L and hypokalemia at 3 mmol/L, normal creatinine at 0.9 mg/dL, elevated blood urea nitrogen (BUN) at 18 mg/dL, elevated total bilirubin at 4.1 mg/dL, elevated aspartate aminotransferase at 134 IU/L and alanine transaminase at 38 IU/L, low alkaline phosphate, elevated lactate dehydrogenase at 2248 IU/L, and undetectable haptoglobin. She was transfused 2 units of pRBCs and 2 units of single-donor platelets. The following morning, her Hgb posttransfusion remained at 7.0 g/dL. She therefore received another unit of pRBCs and was transferred to our institution for further management.

On arrival at our institution (Day 0), she reported ongoing fatigue and passing dark-colored urine. Laboratory values showed worsening anemia suggestive of hemolytic anemia (Table 1). A peripheral smear showed marked macrocytic anemia with numerous teardrop cells and rouleaux, absolute neutropenia with toxic granulation, and marked thrombocytopenia.

**Figure 1:**
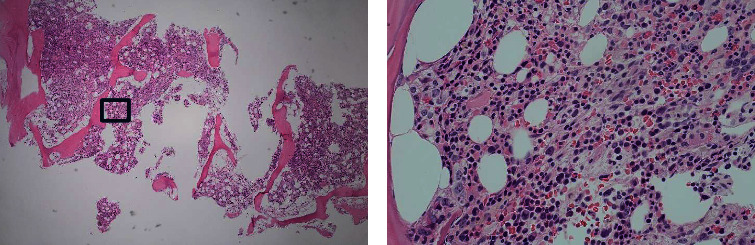
Bone marrow histology in hyperhemolysis syndrome. Hematoxylin and eosin stains of bone marrow core biopsies show increased erythroid-predominant hematopoiesis at (a) 4x and (b) 40x magnifications.

The following day (Day +1), her Hgb continued to drop despite receiving 5 units of pRBC in the past 24 hours. Her Hgb incremented from 4.8 g/dL to 6.1 g/dL with two units of pRBC transfusion only to fall back to 4.7 g/dL after an additional 3 units of pRBC. With no signs of active bleeding, a concern for autoimmune hemolytic anemia was raised, and she was started on 1 mg/kg prednisone daily on Day +1. In addition, due to elevated ferritin levels of 10,258 ng/mL and soluble plasma interleukin-2 receptor of 4364 pg/mL (range: 266.5–1410.4), a bone marrow biopsy was performed on Day +2 to rule out hemophagocytic lymphohistiocytosis. This revealed a hypercellular marrow (80%) with erythroid-predominant trilineage hematopoiesis and erythroid hyperplasia, with minimal hemophagocytosis present (Figures 1(a) and 1(b)).

**Figure 2:**
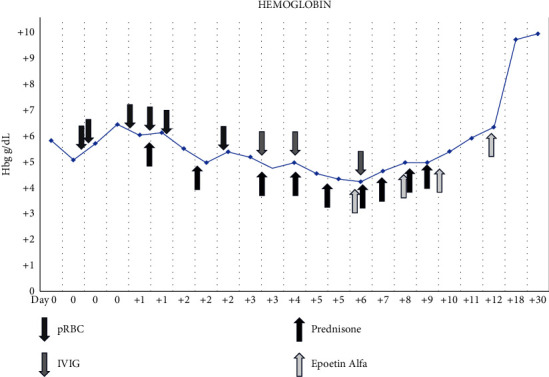
Trends of hemoglobin levels throughout her hospitalization and on discharge.

Testing resulted from a specimen collected on Day 0 revealed alloanti-Jk(a) and alloanti-P1 in plasma reactive at the Coombs phase with polyethylene glycol (PEG) enhancement. The reactivity strength was microscopic for both. A direct antiglobulin test was microscopically positive using a polyspecific reagent (combined IgG + C3d), and monospecific testing was also microscopically reactive for anti-C3d (anti-IgG monospecific testing was negative). An elution was performed on RBCs from this sample which again revealed microscopic reactivity at the Coombs phase with Jk(a)- and P1-positive cells. Notably, she reported feeling worse with each transfusion that she received, and attempts to transfuse pRBC units that were antigen negative for Jk(a) and P1 did not result in an appropriate response in terms of her hemoglobin and hematocrit. Importantly, her Hgb continued to drop to below pretransfusion levels several times following each pRBC transfusion (Figure 2).

A diagnosis of delayed hemolytic transfusion reaction (DHTR) was made, as well as acute hemolytic transfusion reaction (AHTR) during the active hemolysis process occurring on Day +1. The patient was also suspected to have hyperhemolysis syndrome (HS) despite no known history of hemoglobinopathy. Intravenous immunoglobulin (IVIG) at 0.5 g/kg ideal body weight was initiated on Day +3 for four consecutive days, and prednisone at 1 mg/kg was continued. No further blood transfusions were recommended unless she was to develop symptomatic anemia or other evidence of organ hypoperfusion.

A repeat DAT on Day +4 was microscopically positive for IgG and negative for C3d and remained reactive for anti-Jk(a) and anti-P1. In addition, reactivity for Cw (RH8) antigen-positive cells was noted (a rare antigen in the Rh system present in ∼1-2% of donors). An erythropoietin-stimulating agent, epoetin alfa, was initiated on Day +6 and was given every two days until Day +12. She was discharged on Day +12 with an Hgb of 6.0 g/dL with plans to taper off the prednisone dose. On Day +18, her Hgb had improved to 9.2 g/dL. The patient was counselled that if she required future transfusion, she should receive Jk(a)-negative, Cw (RH8)-negative, and P1-negative blood.

## 2. Discussion

HS is a severe hemolytic transfusion reaction in which hemoglobin and hematocrit levels drop much lower than pretransfusion levels, leading to life-threatening anemia. In addition to decreased hemoglobin levels, laboratory findings indicative of HS include evidence of hemolysis such as elevated lactate dehydrogenase, elevated bilirubin, low haptoglobin, and low reticulocyte count. Presenting symptoms often include fever and pain, and cases of both acute and delayed HS have been recorded. Depending on the severity of hemolysis, patients can present with evidence of impaired oxygen delivery and lactic acidosis [1].

The vast majority of patients with this condition have a pre-existing hemoglobinopathy such as sickle cell disease or beta-thalassemia [2]. Although there have been notable cases reported in patients with leukemia or lymphoma [3–6], HS is rarely reported in any other patient population.

HS is particularly dangerous because it involves the destruction of both transfused and autologous red blood cells. Thus, hemoglobin levels can drop well below pretransfusion levels, with additional blood transfusions only accelerating hemolysis. This is opposed to other transfusion reaction syndromes, such as typical delayed hemolytic transfusion reactions (DHTRs), in which only transfused red blood cells (RBCs) are hemolyzed by alloantibodies and autologous RBCs are spared, causing hemoglobin to only drop to its pretransfusion level [3].

Reticulocytopenia is another deleterious effect of HS as opposed to normal hemolytic reactions where the marrow attempts to compensate for the drop in HCT by causing reticulocytosis. It is hypothesized that reticulocytopenia is caused by peripheral destruction of reticulocytes by macrophages via contact lysis and hemophagocytosis. Another proposed mechanism is “bystander hemolysis,” in which antibodies against human leukocyte antigens on donor RBCs and plasma proteins start the cascade that leads to complement activation, which increases destruction of autologous RBCs. This mechanism is supported by the fact that in some cases of HS, no RBC alloantibodies are identified.

Over one third of DHTRs are due to anti-Jk(a), which is known to cause rapid hemolysis that is often fatal. It remains unknown if Jk antibodies are more common in HS [7]. Anti-P1 antibodies are a rarer cause of HS, as in the case of our patient. The P1 phenotype of the RBC surface P1PK complex is found in the majority of people of European or African descent. Anti-P1 antibodies are found in approximately 67% of people without the P1 phenotype but are typically clinically insignificant, as they usually only react in colder temperatures (≤25°C) [8]. The anti-P1 antibodies present in the serum of our patient were found to be reactive at body temperature, suggesting one etiology for the patient's HS after previous transfusions. In addition, reactivity for Cw (RH8) antigen-positive cells was noted in the patient's serum. Anti-Cw is directed against a low incidence antigen present in 1-2% of the population; therefore, the vast majority (98-99%) of donor units are Cw negative, and we do not suspect this antibody as the cause of HS in this patient.

Several potential causative mechanisms were discussed previously, including macrophage hemophagocytosis and “bystander hemolysis.” In support of the activated macrophage hypothesis, recent case reports have indicated that treatment with tocilizumab can result in resolution of hemolysis in refractory or difficult cases [9–11]. Tocilizumab is a humanized monoclonal antibody directed against the interleukin-6 receptor (IL-6R) [12]. IL-6 signaling contributes to macrophage activation [13], and tocilizumab ameliorates deleterious macrophage activation in other disorders [14]. The emergence of new targeted therapeutics such as tocilizumab aimed at the underlying pathogenetic mechanism has the potential to shorten disease duration and improve outcomes in HS. The level of evidence to support the use of these agents in the treatment of HS could be expanded by randomized controlled trials.

## 3. Conclusion

Although HS is well documented in the hemoglobinopathy population, it rarely occurs in the general population. In this patient, the likely triggers for HS were alloantibodies to Jk(a) and P1, which also caused AHTR and DHTR, confounding the clinical presentation. In a patient with no hemoglobinopathy, DHTR is most often suspected with a presentation of chills, flu-like symptoms, headache, fever, and dark amber-colored urine after a blood transfusion. However, HS is an important diagnosis to consider, as further blood transfusions will only worsen symptoms in a patient with severe anemia caused by HS.

## Figures and Tables

**Table 1:** Laboratory values at initial presentation at our institution.

Complete metabolic panel	Range	Result
Sodium	136–144 mmol/L	**135↓**
Potassium	3.3–5.0 mmol/L	^*∗*^
Chloride	102–110 mmol/L	106
Carbon dioxide	20–26 mmol/L	21
Blood urea nitrogen (BUN)	8–24 mg/dL	22
Creatinine	0.57–1.11 mg/dL	**1.2↑**
Glucose	64–128 mg/dL	**189↑**
Calcium	8.4–10.5 mg/dL	8.5
Protein, total	6.5–8.4 g/dL	^*∗*^
Albumin	3.5–5.0 g/dL	**3.2↓**
Bilirubin, total	0.2–1.4 mg/dL	**3.4↑**
Alkaline phosphatase	38–126 U/L	44
Aspartate transaminase (AST)	16–40 U/L	^*∗*^
Alanine transaminase (ALT)	5–60 U/L	41
Anion gap	8–14 mmol/L	8
Lactate dehydrogenase	100–253 U/L	**4233↑**
Magnesium	1.6–2.6 mg/dL	1.9
Phosphorus, inorganic	2.2–4.5 mg/dL	2.3
Procalcitonin	≤0.07 ng/mL	**24.77↑**
Uric acid	2.5–7.5 mg/dL	5.8

Complete blood count	Range	Result

White blood cells	4.30–11.30 k/uL	**1.37↓**
Red blood cells	4.08–5.47 M/uL	**1.59↓**
Hemoglobin	12.6–15.9 g/dL	**5.5↓**
Hematocrit	36.0–49.0%	**16↓**
Mean corpuscular volume (MCV)	81.9–101.0 fL	100.6
Mean corpuscular hemoglobin (MCH)	25.8–33.1 pg	**34.6↑**
Mean corpuscular hemoglobin concentration (MCHC)	31.2–34.5 g/dL	34.4
Red cell distribution width (RDW)	11.5–15.3%	**19.1↑**
Platelet	159–439 k/uL	**10↓**
Immature pit fraction	1.0–11.4%	**19.5↑**
Neutrophil count	2.0–7.4 k/uL	**0.8↓**
Reticulocyte number	47.0–127.0 k/uL	**42↓**
Reticulocytes (%)	1.0–2.6%	2.6
Total lymphocytes (%)	17–50%	30
Total neutrophil (%)	39–73%	57

Other studies	Range	Result

Iron	30–160 ug/dL	**189↑**
Iron-binding capacity	240–450 ug/dL	**213↓**
Transferrin saturation	20–50%sat	**89↑**
Bilirubin direct	0.0–0.5 mg/dL	0.5
Bilirubin, total	0.2–1.4 mg/dL	3.3
Ferritin	12–240 ng/mL	**10,258↑**
Folate, serum	≥5.9 ng/mL	>22.3
Haptoglobin	30–200 mg/dL	**<10↓**

Coagulation	Range	Result

Prothrombin time	12.0–15.5 sec	13.7
International normalized ratio (INR)	Ratio	1.1
Partial thromboplastin time (PTT)	24–35 sec	35
Fibrinogen	150–430 mg/dL	**478↑**
D-dimer	0.0–0.4 ug/mL	**14.4↑**
ADAMTS13 activity	≥61%	70

^*∗*^Unable to quantitate due to hemolysis of the sample. Bold values represent the abnormal values.

## Data Availability

Deidentified data used to support the findings of this study are available from the corresponding author upon request.
